# In-hospital mortality of COVID-19 patients hospitalized with ST-segment elevation myocardial infarction: A meta-analysis

**DOI:** 10.1016/j.ijcha.2022.101151

**Published:** 2022-11-17

**Authors:** Huzaifa Ahmad Cheema, Muhammad Ehsan, Muhammad Ayyan, Abia Shahid, Minaam Farooq, Muhammad Usman Javed, Hassan Mehmood Lak, Malik Qistas Ahmad, Hafeez Ul Hassan Virk, Vladimir Lakhter, Ka Yiu Lee

**Affiliations:** a Department of Medicine, King Edward Medical University, Lahore, Pakistan; b Department of Internal Medicine, Eastern Virginia Medical School, Norfolk, VA, United States; c Section of Clinical Cardiology, Robert and Suzanne Tomsich Department of Cardiovascular Medicine, Cleveland Clinic, Cleveland, OH, United States; d Department of Internal Medicine, University of Pittsburgh Medical Center, Pittsburgh, PA, United States; e Department of Cardiology, Adena Regional Medical Center, Chillicothe, OH, United States; f Cardiology Division, Department of Internal Medicine, Temple University Hospital, Philadelphia, PA, United States; g Swedish Winter Sports Research Centre, Department of Health Sciences, Mid Sweden University, Östersund, Sweden

**Keywords:** ST-segment elevation myocardial infarction, COVID-19, SARS-CoV-2, Mortality

## Abstract

**Introduction:**

Coronavirus Disease 2019 (COVID-19) has been associated with an increased risk of adverse cardiovascular events including arteriovenous thrombosis, myocarditis and acute myocardial injury. Relevant literature to date has reported widely varying estimates of mortality, ranging from approximately 2 to 11 times higher odds of mortality in COVID-19-positive STEMI (ST-segment elevation myocardial infarction) patients. Hence, we conducted this *meta*-analysis to resolve these inconsistencies and assess the impact of COVID-19 infection on mortality and other clinical outcomes in patients presenting with STEMI.

**Methods:**

This *meta*-analysis was registered in PROSPERO (CRD42021297458) and performed according to the Cochrane Handbook for Systematic Reviews of Interventions. PubMed and Embase were searched from inception to November 2021 (updated on April 2022) using a search strategy consisting of terms relating to COVID-19, STEMI, and mortality.

**Results:**

We identified 435 studies through our initial search. After screening according to our eligibility criteria, a total of 11 studies were included. Compared with the non-COVID-19 STEMI patients, the in-hospital mortality rate was higher in COVID-19-positive STEMI patients. Similarly, the risk of cardiogenic shock was higher in the COVID-19-positive patients. Length of hospital stay was longer in STEMI patients with COVID-19.

**Conclusions:**

Our study highlights the necessity for early evaluation of COVID-19 status in all STEMI patients followed by risk stratification, prompt reperfusion and more aggressive management of COVID-19-positive patients. Further research is needed to elucidate the mechanisms behind poorer prognosis in such patients.

## 1 Manuscript

Coronavirus Disease 2019 (COVID-19) has been associated with an increased risk of adverse cardiovascular events including arteriovenous thrombosis, myocarditis and acute myocardial injury.[1], [2], [3] While the outcomes in ST-segment elevation myocardial infarction (STEMI) patients during the COVID-19 pandemic compared with the pre-pandemic era have been investigated,[4] there is a paucity of robust, broadly representative data directly comparing outcomes between STEMI patients with and without a diagnosis of COVID-19. Relevant literature to date has reported widely varying estimates of mortality, ranging from approximately 2 to 11 times higher odds of mortality in COVID-19-positive STEMI patients. Hence, we conducted this *meta*-analysis to resolve these inconsistencies and assess the impact of COVID-19 infection on mortality and other clinical outcomes in patients presenting with STEMI.

This *meta*-analysis was registered in PROSPERO (CRD42021297458) and performed according to the Cochrane Handbook for Systematic Reviews of Interventions.[5] PubMed and Embase were searched from inception to November 2021 (updated on April 2022) using a search strategy consisting of terms relating to COVID-19, STEMI, and mortality (Table S1). We also screened reference lists of relevant articles to procure any missed studies. We included studies that compared our primary outcome, in-hospital mortality, between COVID-19-positive and COVID-19-negative cohorts with primarily out-of-hospital STEMI (Table S2). We excluded all studies that compared the COVID-19 era with the pre-pandemic era without considering the COVID-19 status of STEMI patients. The screening and data extraction processes were independently carried out by two authors. Our secondary outcomes were cardiogenic shock on presentation, door-to-balloon (D2B) time and length of hospital stay. Data were pooled using a random-effects model with odds ratios (ORs) and mean differences (MDs) as effect measures. Publication bias was assessed using Egger’s test for funnel plot asymmetry. RevMan 5.4 and JASP 0.14.1.0 were used for all analyses. Risk-of-bias assessment was conducted using the Newcastle-Ottawa Scale (NOS) (Supplementary Methods).

**Fig. 1:**
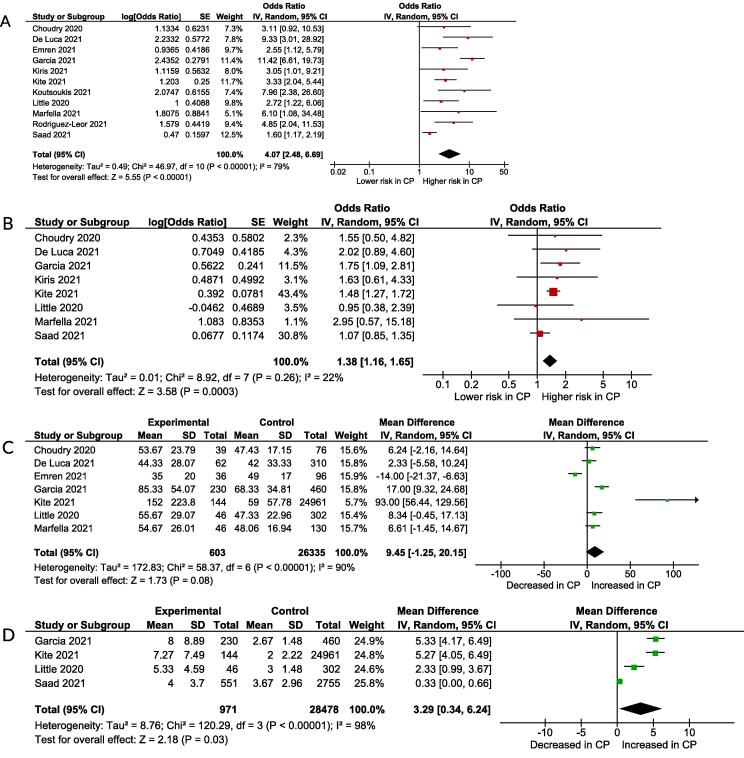
Forest plots displaying **(a)** The odds of in-hospital mortality in COVID-19-positive (CP) vs COVID-19-negative (CN) STEMI patients; **(b)** The odds of cardiogenic shock on presentation in CP vs CN; **(c)** The mean difference for D2B time between CP and CN; **(d)** The mean difference for the length of hospital stay between CP and CN.

We identified 435 studies through our initial search. After screening according to our eligibility criteria, a total of 11 studies were included (Fig. S1). The references for included studies are given in Supplementary References. The characteristics of included participants are presented in Table S3. Most of our studies (n = 6) were of high quality while the most common flaw was the lack of comparability of cohorts (4 studies: Table S4). Compared with the non-COVID-19 STEMI patients, the in-hospital mortality rate was higher in COVID-19-positive STEMI patients (OR 4.07; 95 % CI: 2.48–6.69, I^2^ 79 %; Fig. 1a). Similarly, the risk of cardiogenic shock was higher in the COVID-19-positive patients (OR 1.38; 95 % CI: 1.16–1.65, I^2^ 22 %; Fig. 1b). There was no difference in D2B time between the two cohorts (MD 9.45 min, 95 % CI: −1.25 to 20.15 min, I^2^ 90 %; Fig. 1c). Length of hospital stay, however, was longer in STEMI patients with COVID-19 (MD 3.29 days, 95 % CI: 0.34–6.23 days; I^2^ 97 %; Fig. 1d). We performed subgroup analyses of in-hospital mortality based on the use of unadjusted values versus adjusted values and historical versus concurrent cohort in the included studies (Figs. S2 and S3). No statistically significant difference was observed between the subgroups for both analyses. No publication bias was detected in our primary outcome according to Egger’s test (*P* = 0.352; Fig. S4).

To our knowledge, this is the first comprehensive *meta*-analysis investigating in-hospital mortality in COVID-19-positive STEMI patients. Our results show that the odds of in-hospital mortality are four times higher in these patients than in those without a diagnosis of COVID-19. However, there was no difference in D2B time between the COVID-19 and the non-COVID-19 group.

A previous *meta*-analysis demonstrated that mortality rates in STEMI patients during the COVID-19 pandemic were higher compared with the pre-pandemic era.[4] However, since the studies included in this *meta*-analysis did not assess COVID-19 status, it remains unclear whether the increase in mortality is the result of pandemic-related dynamics, such as patients’ reluctance to seek medical care and overwhelmed health services or the direct effect of COVID-19 infection. Our *meta*-analysis differs from previous ones as it includes an entirely different set of studies by excluding those that did not assess COVID-19 status in patients presenting with STEMI.

COVID-19 is associated with a hyperinflammatory state causing plaque disruption along with a pro-thrombotic milieu.[3], [6] In addition, the angiotensin-converting enzyme-2 (ACE-2) receptor present in cardiomyocytes could be a mediator of direct viral damage to the myocardium.[7] Accordingly, findings from a study have indicated that in the 7 days following COVID-19 diagnosis, the risk of myocardial infarction is roughly increased twofold.[8] Notably, the large increase in in-hospital mortality in our study was consistent regardless of the use of a historical or concurrent cohort. This suggests that COVID-19 infection itself might be a bigger contributor to worse prognosis in STEMI patients than deficient care delivery during the pandemic compared with before the pandemic. Moreover, the concerningly high rate of cardiogenic shock seen in COVID-19 patients is likely a major contributor to their poorer prognosis. Our findings are also in-line with a previous *meta*-analysis that included a few small studies and reported a higher risk of mortality in COVID-19-positive STEMI patients.[9] Of note, D2B time did not differ between the two groups further reinforcing the idea that healthcare restrictions during the pandemic might not play a major role in the large increase in mortality seen in the COVID-19 group. However, this finding should be interpreted with caution as the onset-to-balloon time is a potentially important confounding factor for this that could not be explored well in our review due to lack of data from included studies.

There are some limitations of our study including a high risk of confounding bias due to the observational nature of our included studies. Moreover, there was considerable heterogeneity in our outcomes. We used random-effects models and subgroup analyses to address this but differences in STEMI management protocols and COVID-19 infection rates between different regions may limit the interpretation of our results. Additionally, only one of our studies reported separate data regarding in-hospital STEMI, we therefore only analyzed data mainly on out-of-hospital STEMI. Lastly, there was a lack of long-term outcomes in our studies which could provide more insights into the effects of COVID-19 infection on STEMI patients.

Our study highlights the necessity for early evaluation of COVID-19 status in all STEMI patients followed by risk stratification, prompt reperfusion and more aggressive management of COVID-19-positive patients. Further research is needed to elucidate the mechanisms behind poorer prognosis in such patients.

## Human and animal participants

Research involving human participants and/or animals: No animals or human subjects were used in the current study.

## Informed consents

No informed consents were required for the purpose of the current study.

## Availability of data

The data that support the findings of this study are available from the first author upon reasonable request.

## CRediT authorship contribution statement

**HAC:** Conceptualization, Investigation. **ME:** Conceptualization, Writing- original draft. **MA:** Conceptualization, Formal analysis, Writing- original draft. **AS:** Conceptualization, Data curation, Writing- review & editing. **MF:** Data curation, Writing- original draft. **MUJ:** Data curation, Investigation, Writing- review & editing. **HML:** Formal analysis, Data curation, Writing- review & editing. **MQA:** Data curation, Writing- review & editing. **HHK:** Investigation, Writing- original draft. **VL:** Formal analysis, Data curation, Writing- review & editing. **KYL:** Formal analysis, Investigation, Data curation, Writing- review & editing.

## Declaration of Competing Interest

The authors declare that they have no known competing financial interests or personal relationships that could have appeared to influence the work reported in this paper.
