# Supplementary material for: In-hospital mortality of COVID-19 patients hospitalized with ST-segment elevation myocardial infarction: A meta-analysis
Source: Int J Cardiol Heart Vasc. 2022 Nov 17;43:101151. doi: 10.1016/j.ijcha.2022.101151 (PMC9671642; doi:10.1016/j.ijcha.2022.101151)
Supplement: Supplementary data 1 [file mmc1.docx]

# Supplemental Online Content

**In-hospital mortality of COVID-19 patients hospitalized with ST-segment elevation myocardial infarction: A meta-analysis**

**Authors:**

Huzaifa Ahmad Cheema, MBBS; Muhammad Ehsan, MBBS; Muhammad Ayyan, MBBS; Abia Shahid, MBBS; Minaam Farooq, MBBS; Muhammad Usman Javed, MD; Hassan Mehmood Lak, MD; Malik Qistas Ahmad, MD; Hafeez Ul Hassan Virk, MD; Vladimir Lakhter, DO; Ka Yiu Lee, PhD

# Contents:

**Table S1.** Eligibility criteria for including studies in the review (PECOS)

**Table S2.** Search strategy for Medline (PubMed) which was adapted accordingly for Embase (Ovid)

**Table S3.** Characteristics of included studies

**Table S4.** Quality Assessment of each included cohort study according to Newcastle-Ottawa Scale

**Figure S1.** PRISMA flowchart depicting study selection process

**Figure S2.** Subgroup analysis for in-hospital mortality on the basis of use of unadjusted vs adjusted ORs

**Figure S3.** Subgroup analysis for in-hospital mortality on the basis of use of historical vs concurrent cohorts

**Figure S4.** Funnel plot for in-hospital mortality

**Supplementary Methods**

**Supplementary References**

**Table S1:** **Eligibility criteria for including studies in the review (PECOS)**

| **Category** | **Inclusion and exclusion criteria** |
| --- | --- |
| Participants/population (P) | Patients with primarily out-of-hospital STEMI and a concomitant diagnosis of COVID-19 without any restriction of gender, race or setting will be included. Studies comparing pre-pandemic populations with pandemic populations will be excluded. |
| Exposure (E) | Patients with active COVID-19 infection will be included. |
| Comparator (C) | Patients with STEMI and no active COVID-19 infection will be considered eligible. The comparator period may be concurrent or historical. |
| Outcome (O) | Only studies evaluating all-cause in-hospital mortality will be considered eligible. |
| Studies (S) | All types of comparative observational studies will be included. Non-comparative studies (e.g., case reports, case series) will be excluded. |

**Table S2: Search strategy for Medline (PubMed) which was adapted accordingly for Embase (Ovid)**

| Number | Search terms |
| --- | --- |
| #1 | ("COVID-19" OR "COVID-19"[MeSH Terms] OR "SARS-CoV-2" OR "sars-cov-2"[MeSH Terms] OR "Severe Acute Respiratory Syndrome Coronavirus 2" OR "NCOV" OR "2019 NCOV" OR (("coronavirus"[MeSH Terms] OR "coronavirus" OR "COV") AND 2019/11/01[PDAT] : 3000/12/31[PDAT])) |
| #2 | "st elevation myocardial infarction"[MeSH Terms] OR ("st"[All Fields] AND "elevation"[All Fields] AND "myocardial"[All Fields] AND "infarction"[All Fields]) OR "st elevation myocardial infarction"[All Fields] OR "stemi"[All Fields] |
| #3 | "mortality"[MeSH Terms] OR "mortality"[All Fields] OR "mortalities"[All Fields] OR "mortality"[Subheading] |
| #4 | "hospital mortality"[MeSH Terms] OR ("hospital"[All Fields] AND "mortality"[All Fields]) OR "hospital mortality"[All Fields] OR ("hospital"[All Fields] AND "mortality"[All Fields]) OR "in hospital mortality"[All Fields] |
| #5 | # 3 OR #4 |
| #6 | #1 AND #2 AND #5 |

**Table S3. Characteristics of included studies**

| **Study ID** | **Location** | **Cohort** | **Time period** | **Number of STEMI patients** | **Age (years)^*^** | **Male, n (%)** | **HTN, n (%)** | **Diabetes, n (%)** | **Dyslipidemia, n (%)** | **Smoking, n (%)** | **Prior MI, n (%)** | **Prior PCI, n (%)** | **Door to balloon time (minutes)** | **Cardiogenic shock** | | **In-hospital mortality** | | **ICU admission, n (%)** | **Length of ICU stay (days)** | **Length of hospital stay (days)** |
| --- | --- | --- | --- | --- | --- | --- | --- | --- | --- | --- | --- | --- | --- | --- | --- | --- | --- | --- | --- | --- |
|  |  |  |  |  |  |  |  |  |  |  |  |  |  | **n (%)** | **Adjusted OR (95% CI)** | **n (%)** | **Adjusted OR (95% CI)** |  |  |  |
| Choudry et al, 2020 | UK | CP | 1 March - 20 May 2020 | 39 | 61.7 ± 11.0 | 33 (84.6) | 28 (71.8) | 18 (46.2) | 24 (61.6) | 24 (61.6) | 6 (15.4) | 9 (23.1%) | 53.67±23.79 | 6 (15.4) | NR | 7 (17.9) | NR | 28 (71.8) | NR | NR |
|  |  | CN | 1 March - 20 May 2020 | 76 | 61.7 ± 12.6 | 57 (75.0) | 32 (42.1) | 20 (26.3) | 28 (36.8) | 35 (46.1) | 3 (3.9) | 5 (6.6%) | 47.43±17.15 | 8 (10.5) |  | 5 (6.5) |  | 7 (9.2) | NR | NR |
| Little et al, 2020 | UK | CP | 1 March - 30 April 2020 | 46 | 63 (58–67) | 37 (80.4) | 25 (54.0) | 15 (32.6) | 24 (52.2) | 19 (41.3) | 5 (10.9) | 1 (2.2) | 55.67±29.07 | 6 (13.0) | NR | 10 (21.7) | NR | 15 (32.6) | NR | 5.33±4.59 |
|  |  | CN | 1 March - 30 April 2020 | 302 | 63 (55–72) | 241 (79.8) | 153 (50.7) | 71 (23.5) | 100 (33.1) | 126 (41.7) | 38 (12.6) | 40 (13.3) | 47.33±22.96 | 41 (13.6) |  | 28 (9.3) |  | 28 (9.3) | NR | 3.00±1.48 |
| De Luca et al, 2021 | Europe | CP | 1 March - 30 April 2020 | 62 | 70 (62–76) | 49 (79.0) | 35 (56.5) | 10 (16.1) | 25 (40.3) | 14 (22.6) | 8 (12.9) | 12 (19.4) | 44.33±28.07 | 9 (14.5) | NR | 18 (29.0) | 9.33 (3.01-28.03) | NR | NR | NR |
|  |  | CN | 1 March - 30 April 2020 | 310 | 70 (62–75) | 245 (79.0) | 176 (56.8) | 67 (21.6) | 125 (40.3) | 109 (35.2) | 29 (9.4) | 49 (15.8) | 42.00±33.33 | 24 (7.7) |  | 17 (5.5) |  | NR | NR | NR |
| Emren et al, 2021 | Turkey | CP | December 2020 - March 2021 | 36 | 64.0±10.0 | 26 (72.2) | 25 (69.0) | 18 (50.0) | 14 (39.0) | 20 (56.0) | NR | 5 (14.0) | NR | NR | NR | 15 (42.0) | NR | NR | NR | NR |
|  |  | CN | December 2020 - March 2021 | 96 | 60.0±13.0 | 72 (75.0) | 42 (42.0) | 27 (27.0) | 27 (27.0) | 66 (67.0) | NR | 15 (15.0) | NR | NR |  | 21 (21.0) |  | NR | NR | NR |
| Garcia et al, 2021 | Canada, USA | CP | 1 January - 6 December 2020 | 230 | NR | 164 (71.0) | 166 (73.0) | 103 (46) | 101 (46.0) | 94 (44) | 26 (13 | 28 (13) | 85.33±54.07 | 36 (18) | NR | 73 (33.0) | NR | NR | 3 (1–10) | 8.00±8.89 |
|  |  | CN | January 2015 - December 2019 | 460 | NR | 313 (68.0) | 317 (69.0) | 130 (28) | 277 (60.0) | 273 (59) | 111 (24) | 118 (26) | 68.33±34.81 | 44 (10) |  | 18 (4.0) |  | NR | NR | 2.67±1.48 |
| Kiris et al, 2021 | Turkey | CP | 11 March - 15 May 2020 | 59 | 66.1 ± 12.0 | 39 (66.1) | 29 (49) | 16 (27) | NR | 20 (34) | NR | NR | NR | 12 (20) | NR | 13 (22) | NR | NR | NR | NR |
|  |  | CN | 11 March - 15 May 2020 | 59 | 68.1 ± 11.8 | 38 (64.4) | 30 (51) | 21 (36) | NR | 15 (25) | NR | NR | NR | 8 (14) |  | 5 (9) |  | NR | NR | NR |
| Kite et al, 2021 | Europe, Asia, South America, Africa, North America | CP | 1 March - 31 July 2020 | 144 | 63.1 ± 12.6 | 112 (77.8) | 92/142 (64.8) | 49 (34.0) | 58/126 (46.0) | 39/123 (31.7) | 23/140 (16.4) | 20 (13.9) | 152.00±223.80 | 29 (20.1) | 1.48 (1.27–1.72) | 33 (22.9) | 3.33 (2.04–5.42) | 66 (45.8) | NR | 7.27±7.49 |
|  |  | CN | 1 April 2018 - 31 March 2019 | 24,961 | 65.6 ± 13.4 | 17972 (72.2) | 9,456 (44.8) | 4,926 (20.9) | 6,039 (28.9) | 7,645 (33.7) | 2747 (13.0) | 2129 (10.2) | 59.00±57.78 | 1,898/21,972 (8.7) |  | 1,232/21,675 (5.7) |  | NR | NR | 2.00±2.22 |
| Koutsoukis et al, 2021 | France | CP | 1 April - 22 April 2020 | 17 | 63.4 ± 13.2 | 12 (70) | NR | NR | NR | NR | NR | NR | NR | 8 (47.1)^†^ | NR | 7 (41.2) | NR | NR | NR | NR |
|  |  | CN | 1 April - 22 April 2020 | 99 | 66.8 ± 13.9 | 67 (67) | NR | NR | NR | NR | NR | NR | NR | 22 (22.2)^†^ |  | 8 (8.1) |  | NR | NR | NR |
| Marfella et al, 2021 | Italy | CP | February - November 2020 | 46 | 56.13 ± 6.21 | 31 (67.4) | 18 (39.1) | 8 (17.4) | 7 (15.2) | 3 (6.5) | NR | NR | 54.67±26.01 | 3 (6.5) | NR | 4 (8.7) | NR | NR | NR | NR |
|  |  | CN | February - November 2020 | 130 | 68.43 ± 6.46 | 86 (66.2) | 72 (55.4) | 38 (29.2) | 30 (23.7) | 39 (29.2) | NR | NR | 48.06±16.94 | 3 (2.3) |  | 2 (1.5) |  | NR | NR | NR |
| Rodriguez-Leor et al, 2021 | Spain | CP | 14 March - 30 April 2020 | 91 | 64.8±11.8 | 76/90 (84.4) | 47 (51.7) | 21 (23.1) | 44 (48.4) | 17(18.7) | NR | NR | NR | 9 (9.9)^‡^ | NR | 21 (23.1) | 4.85 (2.04- 11.51) | NR | NR | NR |
|  |  | CN | 14 March - 30 April 2020 | 919 | 62.5±13.1 | 717/915 (78.4) | 489 (53.3) | 192/917 (20.9) | 429/915 (46.9) | 415/913 (45.5) | NR | NR | NR | 35 (3.8)^‡^ |  | 52 (5.7) |  | NR | NR | NR |
| Saad et al, 2021 | USA | CP | 1 January - 31 December 2020 | 551 | NR | 385 (69.9%) | 435 (78.9%) | 263 (47.7%) | 364 (66.1%) | 89 (16.2%) | 0 | 3 (0.5%) | NR | 101 (18.3%) | 1.07 (0.85 to 1.36) | 84 (15.2%) | 1.60 (1.17-2.19) | NR* | 1.0 (0.0, 3.0)- median | 4.00±3.70 |
|  |  | CN | 1 January - 31 December 2019 | 2755 | NR | 1939 (70.4%) | 2207 (80.1%) | 1345 (48.8%) | 1845 (67%) | 451 (16.4%) | 2 (0.1%) | 31 (1.1%) | NR | 476 (17.3%) |  | 308 (11.2%) |  | NR | 1.0 (0.0, 3.0) | 3.67±2.96 |
| STEMI = ST elevation myocardial infarction; HTN = hypertension; MI = myocardial infarction; PCI = percutaneous coronary angioplasty; OR = odds ratio; CP = COVID-19-positive; CN = COVID-19-negative; NR = not reported | | | | | | | | | | | | | | | | | | | | |
| *Values reported as mean ± SD or median (IQR); † cardiogenic shock or heart failure; ‡cardiogenic shock post-PCI | | | | | | | | | | | | | | | | | | | | |

**Table S4: Quality Assessment of each included cohort study according to Newcastle-Ottawa Scale**

|  | **Selection** | | | | **Comparability** | | **Outcome** | | |  |
| --- | --- | --- | --- | --- | --- | --- | --- | --- | --- | --- |
| **Study** | **Representativeness of the exposed cohort** | **Selection of the non-exposed cohort** | **Ascertainment of exposure** | **Demonstration that outcome**  **of interest was not present at the start of**  **study** | **Controls for the most important risk factors** | **Controls for other risk factors** | **Assessment of outcome** | **Was follow up long enough for outcomes to occur** | **Adequacy of follow up of cohorts** | **Total quality score** |
| Choudry et al, 2020[1] | 1 | 1 | 1 | 1 | 0 | 0 | 1 | 1 | 1 | **7** |
| Little et al, 2020[2] | 1 | 1 | 1 | 1 | 0 | 0 | 1 | 1 | 1 | **7** |
| De Luca et al, 2021[3] | 1 | 1 | 1 | 1 | 1 | 1 | 1 | 1 | 1 | **9** |
| Emren et al, 2021[4] | 1 | 1 | 1 | 1 | 0 | 0 | 1 | 1 | 1 | **7** |
| Garcia et al, 2021[5] | 1 | 1 | 1 | 1 | 1 | 1 | 1 | 1 | 1 | **9** |
| Kiris et al, 2021[6] | 1 | 1 | 1 | 1 | 1 | 1 | 1 | 1 | 1 | **9** |
| Kite et al, 2021[7] | 1 | 1 | 1 | 1 | 1 | 1 | 1 | 1 | 1 | **9** |
| Koutsoukis et al, 2021[8] | 1 | 1 | 1 | 1 | 0 | 0 | 1 | 1 | 1 | **7** |
| Marfella et al, 2021[9] | 1 | 1 | 1 | 1 | 1 | 1 | 0 | 1 | 1 | **8** |
| Rodriguez–Leor et al, 2021[10] | 1 | 1 | 1 | 1 | 1 | 1 | 1 | 1 | 1 | **9** |
| Saad et al, 2021[11] | 1 | 1 | 1 | 1 | 1 | 1 | 1 | 1 | 1 | **9** |

**Figure S1: PRISMA flowchart depicting study selection process**


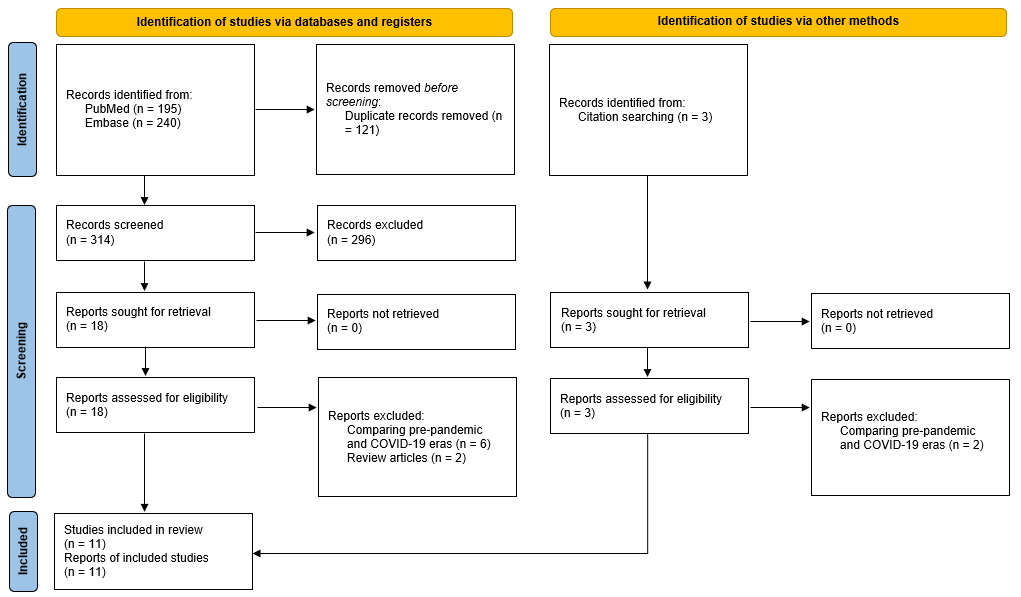


**Figure S2: Subgroup analysis for in-hospital mortality on the basis of use of unadjusted vs adjusted ORs**


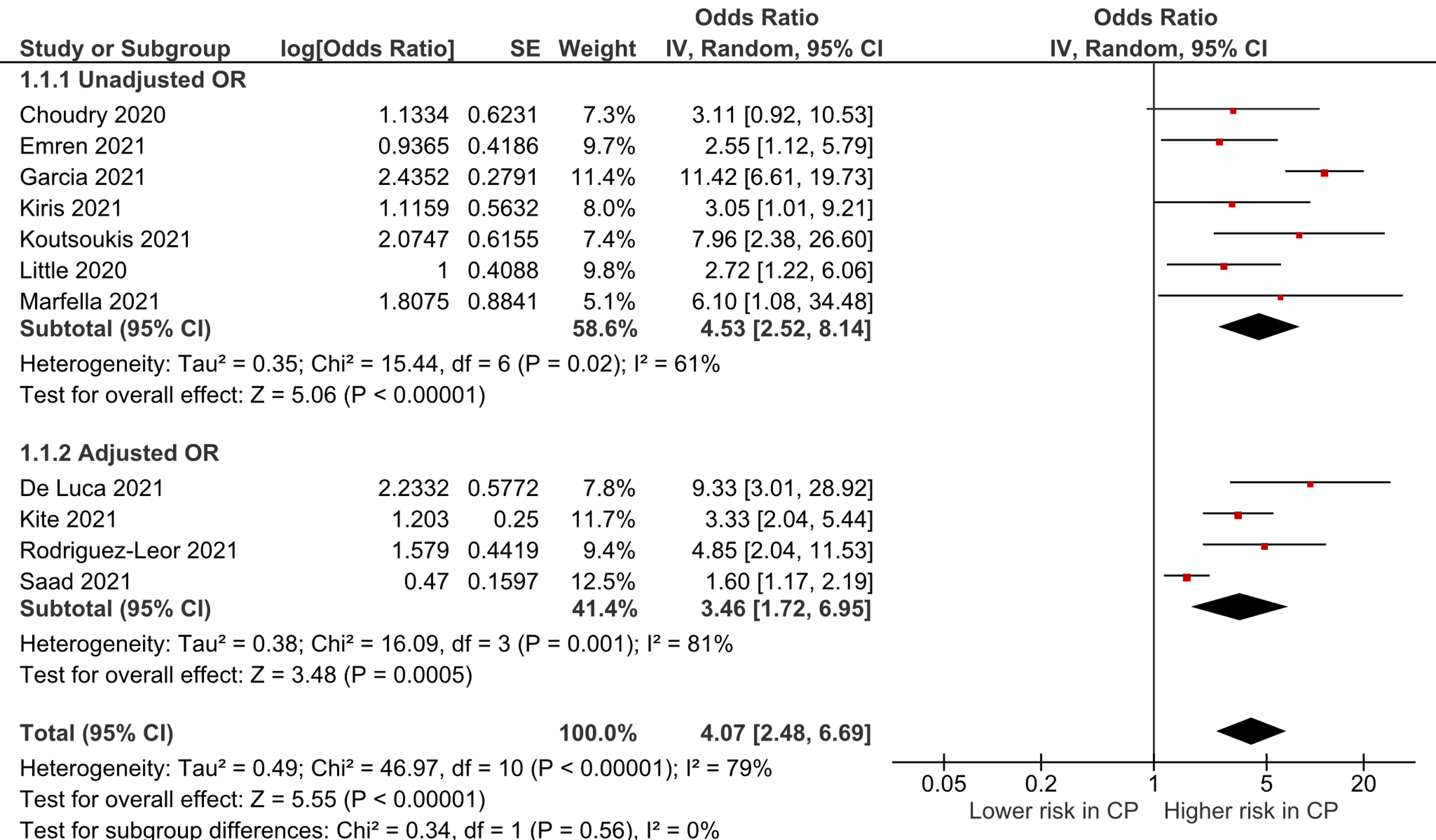


**Figure S3:** **Subgroup analysis for in-hospital mortality on the basis of use of historical vs concurrent cohorts**

**
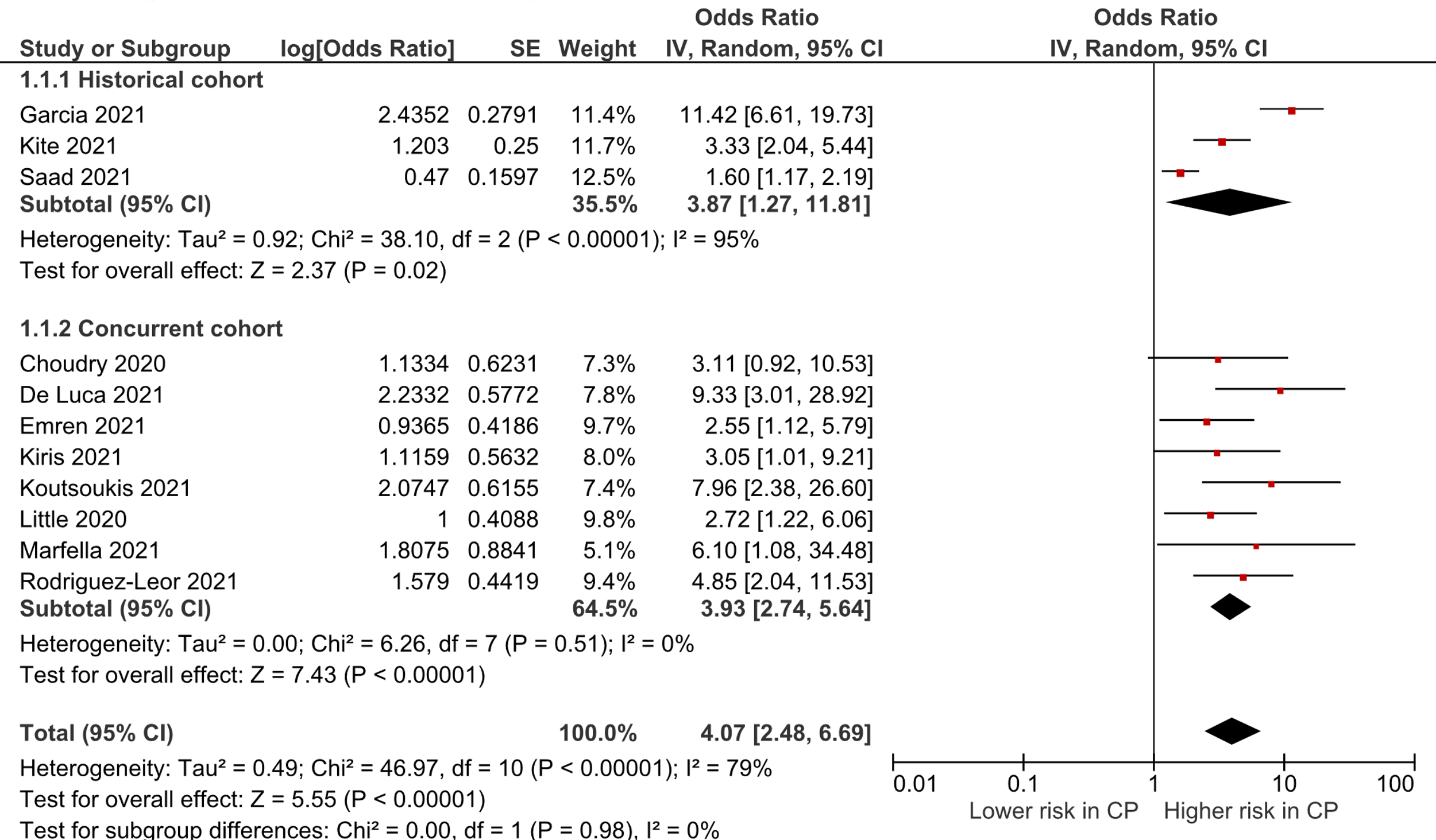
**

**Figure S4. Funnel plot for in-hospital mortality**

**
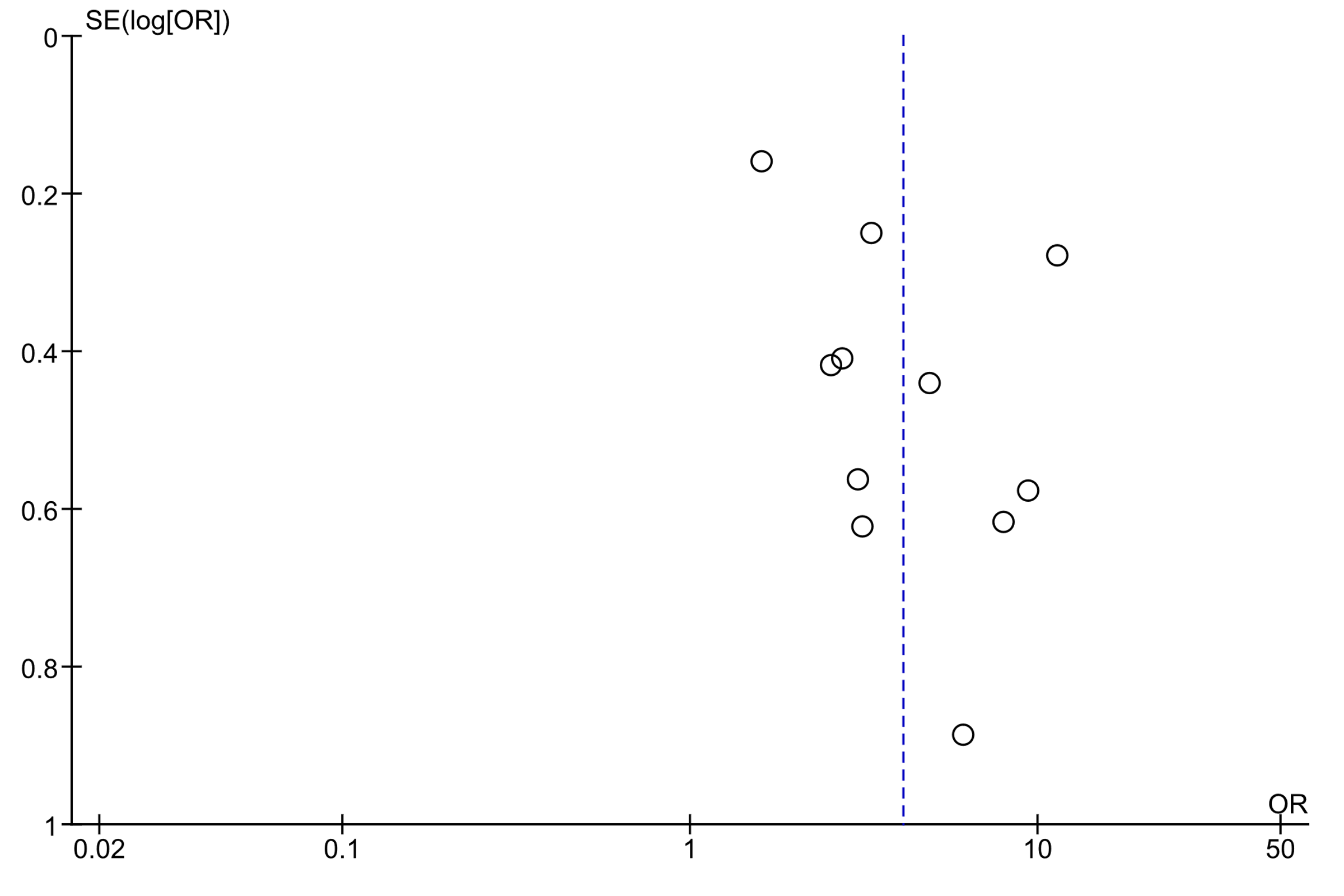
**

**Supplementary Methods**

### Selection of studies

Mendeley Desktop 1.19.8 (Mendeley Ltd**)** was used to remove duplicates from all the obtained literature. After de-duplication, two reviewers independently screened the titles and abstracts of the studies, and then full texts of the potentially relevant articles to retrieve the studies fitting our eligibility criteria. Any discrepancies between the reviewers were resolved through discussion with a third reviewer.

**Risk of bias in individual studies:**

For the assessment of the quality of the observational studies, we used the Newcastle-Ottawa Scale (NOS). The studies were judged by point allocation in three domains with a total of 9 points being the highest possible quality: 1) Selection of study groups (0-4 points), 2) Comparability of cohorts (0-2 points), 3) Establishment of the outcome of interest (0-3 points). Two authors judged the quality of the included studies independently and any disagreement between them was resolved, first by discussion and then by a third author.

**Data collection**

Two reviewers independently extracted the data from the included studies into a pre-piloted structured Excel spreadsheet. Any discrepancies between them were checked by a third reviewer. The data was extracted on the PECOS elements which included the population characteristics (e.g., age, comorbidities, history of smoking), exposure details, comparator details, outcome variables (primary outcome: all-cause in-hospital mortality; secondary outcomes: cardiogenic shock on presentation, door-to-balloon time and length of hospital stay) and study characteristics (e.g., first author name, year of publication, sample size, the name of the country of the recruited patient(s)).

If not reported, mean and SDs were estimated using the methods described by Wan et al.[12] using the study sample size, median, and interquartile range.

### Assessment of heterogeneity

For each synthesis, we calculated the Chi^2^ test and I^2^ statistic to detect the presence of heterogeneity and quantify it, respectively. We interpreted I^2^ values according to the *Cochrane Handbook for Systematic Reviews of Interventions*,[13] section 10.10. *P* < 0.10 was considered statistically significant for the Chi^2^ test.

**Supplementary References**

**References to included studies**

[1] F.A. Choudry, S.M. Hamshere, K.S. Rathod, M.M. Akhtar, R.A. Archbold, O.P. Guttmann, S. Woldman, A.K. Jain, C.J. Knight, A. Baumbach, A. Mathur, D.A. Jones, High Thrombus Burden in Patients With COVID-19 Presenting With ST-Segment Elevation Myocardial Infarction, J. Am. Coll. Cardiol. 76 (2020) 1168–1176. https://doi.org/10.1016/j.jacc.2020.07.022.

[2] C.D. Little, T. Kotecha, L. Candilio, R.J. Jabbour, G.B. Collins, A. Ahmed, M. Connolly, R. Kanyal, O.M. Demir, L.O. Lawson, B. Wang, S. Firoozi, J.C. Spratt, D. Perera, P. MacCarthy, M. Dalby, A. Jain, S.J. Wilson, I. Malik, R. Rakhit, COVID-19 pandemic and STEMI: pathway activation and outcomes from the pan-London heart attack group, Open Hear. 7 (2020) e001432. https://doi.org/10.1136/openhrt-2020-001432.

[3] G. De Luca, N. Debel, M. Cercek, L.O. Jensen, M. Vavlukis, L. Calmac, T. Johnson, G.R. Ferrer, V. Ganyukov, W. Wojakowski, T. Kinnaird, C. von Birgelen, Y. Cottin, A. IJsselmuiden, B. Tuccillo, F. Versaci, K.-J. Royaards, J. Ten Berg, M. Laine, M. Dirksen, M. Siviglia, G. Casella, P. Kala, J.L. Díez Gil, A. Banning, V. Becerra, C. De Simone, A. Santucci, X. Carrillo, A. Scoccia, G. Amoroso, A.W. van’t Hof, T. Kovarnik, G. Tsigkas, J. Mehilli, G. Gabrielli, X.F. Rios, N. Bakraceski, S. Levesque, G. Cirrincione, V. Guiducci, M. Kidawa, L. Spedicato, L. Marinucci, P. Ludman, F. Zilio, G. Galasso, E. Fabris, M. Menichelli, A. Garcia-Touchard, S. Manzo, G. Caiazzo, J. Moreu, J.S. Forés, L. Donazzan, L. Vignali, R. Teles, E. Benit, P. Agostoni, F.B. Ojeda, H. Lehtola, S. Camacho-Freiere, A. Kraaijeveld, Y. Antti, M. Boccalatte, P. Deharo, I.L. Martínez-Luengas, B. Scheller, E. Varytimiadi, R. Moreno, G. Uccello, B. Faurie, A. Gutierrez Barrios, M. Milewski, E. Bruwiere, P. Smits, B. Wilbert, F.S. Di Uccio, G. Parodi, E. Kedhi, M. Verdoia, Impact of SARS-CoV-2 positivity on clinical outcome among STEMI patients undergoing mechanical reperfusion: Insights from the ISACS STEMI COVID 19 registry, Atherosclerosis. 332 (2021) 48–54. https://doi.org/10.1016/j.atherosclerosis.2021.06.926.

[4] Z. Emren, R. Gediz, F. Levent, S. Emren, O. Senoz, Comparison of clinical and angiographic results in COVID-19–positive and –negative patients undergoing primary coronary intervention due to ST-elevation myocardial infarction, Int. J. Cardiovasc. Acad. 7 (2021) 78. https://doi.org/10.4103/ijca.ijca_18_21.

[5] S. Garcia, P. Dehghani, C. Grines, L. Davidson, K.R. Nayak, J. Saw, R. Waksman, J. Blair, B. Akshay, R. Garberich, C. Schmidt, H.Q. Ly, S. Sharkey, N. Mercado, C.E. Alfonso, N. Misumida, D. Acharya, M. Madan, A.M. Hafiz, N. Javed, J. Shavadia, J. Stone, M.C. Alraies, W. Htun, W. Downey, B.A. Bergmark, J. Ebinger, T. Alyousef, H. Khalili, C.-W. Hwang, J. Purow, A. Llanos, B. McGrath, M. Tannenbaum, J. Resar, R. Bagur, P. Cox-Alomar, A.C. Stefanescu Schmidt, L.A. Cilia, F.A. Jaffer, M. Gharacholou, M. Salinger, B. Case, A. Kabour, X. Dai, O. Elkhateeb, T. Kobayashi, H.-H. Kim, M. Roumia, F. V Aguirre, J. Rade, A.-Y. Chong, H.M. Hall, S. Amlani, A. Bagherli, R.A.G. Patel, D.A. Wood, F.G. Welt, J. Giri, E. Mahmud, T.D. Henry, Initial Findings From the North American COVID-19 Myocardial Infarction Registry., J. Am. Coll. Cardiol. 77 (2021) 1994–2003. https://doi.org/10.1016/j.jacc.2021.02.055.

[6] T. Kiris, E. Avci, T. Ekin, D.E. Akgün, M. Tiryaki, A. Yidirim, K. Hazir, B. Murat, M. Yeni, R. Altindag, S. Gül, B. Arik, T. Güzel, S. Murat, A. Oz, M. Karabacak, Z. Aktas, T. Yildirim, B. Kilicaslan, A.O. Ergene, Impact of COVID-19 outbreak on patients with ST-segment elevation myocardial ınfarction (STEMI) in Turkey: results from TURSER study (TURKISH St-segment elevation myocardial ınfarction registry), J. Thromb. Thrombolysis. (2021) 1–14. https://doi.org/10.1007/s11239-021-02487-3.

[7] T.A. Kite, P.F. Ludman, C.P. Gale, J. Wu, A. Caixeta, J. Mansourati, M. Sabate, P. Jimenez-Quevedo, L. Candilio, P. Sadeghipour, A.M. Iniesta, S.P. Hoole, N. Palmer, A. Ariza-Solé, A. Namitokov, H.H. Escutia-Cuevas, F. Vincent, O. Tica, M. Ngunga, I. Meray, A. Morrow, M.M. Arefin, S. Lindsay, G. Kazamel, V. Sharma, A. Saad, G. Sinagra, F.A. Sanchez, M. Roik, S. Savonitto, M. Vavlukis, S. Sangaraju, I.S. Malik, S. Kean, N. Curzen, C. Berry, G.W. Stone, B.J. Gersh, A.H. Gershlick, International Prospective Registry of Acute Coronary Syndromes in Patients With COVID-19, J. Am. Coll. Cardiol. 77 (2021) 2466–2476. https://doi.org/10.1016/j.jacc.2021.03.309.

[8] A. Koutsoukis, C. Delmas, F. Roubille, L. Bonello, G. Schurtz, S. Manzo-Silberman, E. Puymirat, M. Elbaz, F. Bouisset, P.-A. Meunier, F. Huet, F. Paganelli, M. Laine, G. Lemesle, N. Lamblin, P. Henry, V. Tea, R. Gallet, E. Teiger, R. Huguet, D. Fard, P. Lim, Acute Coronary Syndrome in the Era of SARS-CoV-2 Infection: A Registry of the French Group of Acute Cardiac Care., CJC Open. 3 (2021) 311–317. https://doi.org/10.1016/j.cjco.2020.11.003.

[9] R. Marfella, P. Paolisso, C. Sardu, L. Palomba, N. D’Onofrio, A. Cesaro, M. Barbieri, M.R. Rizzo, F.C. Sasso, L. Scisciola, F. Turriziani, M. Galdiero, D. Pignataro, F. Minicucci, M.C. Trotta, M. D’Amico, C. Mauro, P. Calabrò, M.L. Balestrieri, G. Signioriello, E. Barbato, M. Galdiero, G. Paolisso, SARS-COV-2 colonizes coronary thrombus and impairs heart microcirculation bed in asymptomatic SARS-CoV-2 positive subjects with acute myocardial infarction, Crit. Care. 25 (2021) 217. https://doi.org/10.1186/s13054-021-03643-0.

[10] O. Rodriguez-Leor, A.B. Cid Alvarez, A. Pérez de Prado, X. Rossello, S. Ojeda, A. Serrador, R. López-Palop, J. Martin-Moreiras, J.R. Rumoroso, A. Cequier, B. Ibáñez, I. Cruz-González, R. Romaguera, R. Moreno, In-hospital outcomes of COVID-19 ST-elevation myocardial infarction patients., EuroIntervention J. Eur. Collab. with Work. Gr. Interv. Cardiol. Eur. Soc. Cardiol. 16 (2021) 1426–1433. https://doi.org/10.4244/EIJ-D-20-00935.

[11] M. Saad, K.F. Kennedy, H. Imran, D.W. Louis, E. Shippey, A. Poppas, K.E. Wood, J.D. Abbott, H.D. Aronow, Association Between COVID-19 Diagnosis and In-Hospital Mortality in Patients Hospitalized With ST-Segment Elevation Myocardial Infarction, JAMA. 326 (2021) 1940. https://doi.org/10.1001/jama.2021.18890.

**Additional references**

[12] X. Wan, W. Wang, J. Liu, T. Tong, Estimating the sample mean and standard deviation from the sample size, median, range and/or interquartile range, BMC Med. Res. Methodol. 14 (2014) 135. https://doi.org/10.1186/1471-2288-14-135.

[13] J.P.T. Higgins, J. Thomas, J. Chandler, M. Cumpston, T. Li, M.J. Page, V.A. Welch, eds., Cochrane Handbook for Systematic Reviews of Interventions, 2nd ed., Wiley Blackwell, Hoboken, New Jersey, 2019. https://doi.org/10.1002/9781119536604.
